# Prevalence of cervical intraepithelial neoplasia grades II/III and cervical cancer in patients with cytological diagnosis of atypical squamous cells when high-grade intraepithelial lesions (ASC-H) cannot be ruled out

**DOI:** 10.1590/S1516-31802009000500007

**Published:** 2010-02-03

**Authors:** Andréa Cytryn, Fábio Bastos Russomano, Maria José de Camargo, Lucília Maria Gama Zardo, Nilza Maria Sobral Rebelo Horta, Rachel de Carvalho Silveira de Paula Fonseca, Maria Aparecida Tristão, Aparecida Cristina Sampaio Monteiro

**Affiliations:** I MD, MSc. Medical colpocopist, Hospital Geral de Ipanema, Rio de Janeiro, Brazil.; II MD, PhD, MSc. Head of Cervical Pathology Service, Instituto Fernandes Figueira (IFF), Fundação Instituto Oswaldo Cruz (Fiocruz), Rio de Janeiro, Brazil.; III MD, MSc. Cytopathologist in the Serviço Integrado de Tecnologia em Citopatologia (Sitec), Instituto Nacional de Câncer (INCA), Rio de Janeiro, Brazil.; IV MD. Specialist in Anatomical Pathology and Cytopathology in the Serviço Integrado de Tecnologia em Citopatologia (Sitec), Instituto Nacional de Câncer (INCA), Rio de Janeiro, Brazil.; V MD, MSc. Pathologist in the Anatomical Pathology Department, Instituto Fernandes Figueira (IFF), Fundação Instituto Oswaldo Cruz (Fiocruz), Rio de Janeiro, Brazil.; VI MD, MSc. Colposcopist in the Cervical Pathology Service, Instituto Fernandes Figueira (IFF), Fundação Instituto Oswaldo Cruz (Fiocruz), Rio de Janeiro, Brazil.

**Keywords:** Cytology, Colposcopy, Cervical intraepithelial neoplasia, Uterine cervical neoplasm, Cervix uteri, Citologia, Colposcopia, Neoplasia intra-epitelial cervical, Neoplasias do colo do útero, Colo do útero

## Abstract

**CONTEXT AND OBJECTIVE:**

The latest update of the Bethesda System divided the category of atypical squamous cells of undetermined significance (ASCUS) into ASC-US (undetermined significance) and ASC-H (high-grade intraepithelial lesion cannot be ruled out). The aims here were to measure the prevalence of pre-invasive lesions (cervical intraepithelial neoplasia, CIN II/III) and cervical cancer among patients referred to Instituto Fernandes Figueira (IFF) with ASC-H cytology, and compare them with ASC-US cases.

**DESIGN AND SETTING:**

Cross-sectional study with retrospective data collection, at the IFF Cervical Pathology outpatient clinic.

**METHODS:**

ASCUS cases referred to IFF from November 1997 to September 2007 were reviewed according to the 2001 Bethesda System to reach cytological consensus. The resulting ASC-H and ASC-US cases, along with new cases, were analyzed relative to the outcome of interest. The histological diagnosis (or cytocolposcopic follow-up in cases without such diagnosis) was taken as the gold standard.

**RESULTS:**

The prevalence of CIN II/III in cases with ASC-H cytology was 19.29% (95% confidence interval, CI, 9.05-29.55%) and the risk of these lesions was greater among patients with ASC-H than with ASC-US cytology (prevalence ratio, PR, 10.42; 95% CI, 2.39-45.47; P = 0.0000764). Pre-invasive lesions were more frequently found in patients under 50 years of age with ASC-H cytology (PR, 2.67; 95% CI, 0.38-18.83); P = 0.2786998). There were no uterine cervical cancer cases.

**CONCLUSION:**

The prevalence of CIN II/III in patients with ASC-H cytology was significantly higher than with ASC-US, and division into ASC diagnostic subcategories had good capacity for discriminating the presence of pre-invasive lesions.

## RESUMO

**CONTEXTO E OBJETIVO:**

A última atualização do Sistema Bethesda dividiu a categoria de células escamosas atípicas de significado indeterminado (ASCUS) em ASC-US (de significado indeterminado) e ASC-H (quando não se pode excluir lesão intra-epitelial de alto grau). Os objetivos deste estudo foram medir a prevalência da lesão pré-invasiva (Neoplasia Intra-epitelial Cervical, NIC II/III) e câncer cervical, de pacientes que foram encaminhadas ao Instituto Fernandes Figueira (IFF), com citologia ASC-H e compará-la com os casos ASC-US.

**TIPO DE ESTUDO E LOCAL:**

Estudo transversal com coleta de dados retrospectiva, que ocorreu no ambulatório de Patologia Cervical do IFF.

**MÉTODOS:**

Casos com diagnóstico de ASCUS recebidos no IFF entre novembro de 1997 a setembro de 2007, foram revisados de acordo com o Sistema Bethesda 2001 até um diagnóstico de consenso. Os casos ASC-H e ASC-US resultantes desta revisão, e os casos novos, foram analisados em relação ao desfecho de interesse. Consideramos padrão-ouro tanto a histologia como, nos casos sem diagnóstico histológico, o acompanhamento cito-colposcópico.

**RESULTADOS:**

A prevalência de NIC II/III na citologia ASC-H foi de 19,29% (intervalo de confiança, IC 95% 9,05-29,55%) e o risco destas lesões foi maior entre as pacientes com citologia ASC-H comparado às pacientes com citologia ASC-US (razão de prevalência, RP = 10,42; IC 95% 2,39-45,47; P = 0,0000764). A lesão pré-invasiva na citologia ASC-H foi mais frequente abaixo dos 50 anos (RP = 2,67; IC 95% 0,38-18,83), P = 0,2786998. Não houve casos de câncer do colo do útero.

**CONCLUSÕES:**

A prevalência de NIC II/III em pacientes com citologia ASC-H foi significativamente mais alta que a de ASC-US, e a divisão em subcategorias do diagnóstico ASC se mostrou com boa capacidade para discriminar a presença de lesões pré invasivas.

## INTRODUCTION

Several cervicovaginal cytological classification systems have been suggested since Papanicolaou’s classification, with the aim of screening for precursor lesions of cervical cancer. The classification system most used worldwide is American and known as the Bethesda System. Among the abnormalities in squamous tissue, the first version of this classification introduced the term atypical squamous cells of undetermined significance (ASCUS)^1^ to represent a cytological category that did not conform to a normal examination, but did not present all the abnormalities that would be required for interpretation as a squamous intraepithelial lesion (SIL).^2^ The initial proposal for managing such cases was that they should be referred for colposcopy. However, with the backing of several authors, it was observed that a large proportion of these women did not present pre-invasive lesions.^2^

Faced with these arguments, the Bethesda System was reviewed and modified in 1991. In accordance with this second consensus, pathologists were encouraged to specify the type of abnormality observed on the ASCUS diagnostic chart, in terms of whether they led to neoplastic or reactional processes.^3^

The ASCUS/LSIL Triage Study (ALTS) Group conducted a large multicenter randomized clinical trial to establish the best initial strategy for cases with a cytological diagnosis of ASCUS and LSIL (low-grade SIL).^4^ The changes made in the latest review of the Bethesda System in 2001 were that the ASCUS subcategory in which reactional processes were favored was dropped^5^ and the category of atypical squamous cells (ASC) was subdivided into atypical squamous cells of undetermined significance (ASC-US) and atypical squamous cells for which high-grade intraepithelial lesions cannot be ruled out (ASC-H). This last diagnosis represents 5-10% of ASC diagnoses, but with greater likelihood of the presence of pre-invasive lesions.^6^

The criteria for diagnosing ASC-H are varied. This diagnosis is defined as the presence of cell abnormalities that are similar to high-grade lesions but which lack the definite criteria for such lesions.^7^ The cytomorphological criteria for ASC-US are similar to the ones used for ASCUS.^8^^,^^9^

The higher prevalence of pre-invasive lesions, i.e. cervical intraepithelial neoplasia grade II or III (CIN II/III) according to Richart,^10^ in patients with ASC-H diagnosis in relation to those with a diagnosis of ASC-US, justifies immediate referral for colposcopy.^6^^,^^7^^,^^11^^,^^12^

Cross-sectional studies evaluating the prevalence of CIN II and III among individuals with ASC-H cytology have found values between 26 and 68%.^13^^,^^14^^,^^15^^,^^16^^,^^17^^,^^18^^,^^19^ There is some evidence that this prevalence is lower after the menopause.^15^^,^^17^^,^^20^^,^^21^

## OBJECTIVES

The objectives of this study were to determine the prevalence of CIN II and III and cervical cancer among patients with ASC-H cytology in our setting, to analyze this prevalence pre and postmenopausally and to evaluate the risk of CIN II and III in cases with ASC-H cytology in relation to cases with ASC-US cytology.

## MATERIALS AND METHODS

This was a cross-sectional study among women using the Brazilian national health system (Sistema Único de Saúde, SUS) in the city of Rio de Janeiro, with sampling of retrospective data, and the inclusion of cases accepted by the Cervical Pathology Sector of Instituto Fernandes Figueira, Fundação Oswaldo Cruz (IFF/Fiocruz). This study was approved by the institution’s Ethics Committee in May 2007.

**Figure 1.**
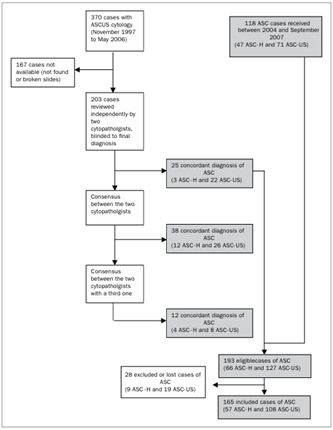
Demonstrative flow chart for the identification and inclusion of cases. The cases identified as atypical squamous cells for which high grade squamous intraepithelial lesions cannot be ruled out (ASC-H) or as atypical squamous cells of undetermined significance (ASC-US) are shown in gray.

Cases were recruited from two sources: (1) cases from the sector’s database that were diagnosed and identified as ASCUS and admitted between November 1997 and May 2006, with follow-up and reclassification in accordance with the 2001 Bethesda System; and (2) new cases of ASC-H and ASC-US cytological diagnoses that were admitted between 2004 and September 2007 (Figure 1).

Out of the 370 cases with an initial diagnosis of ASCUS that were identified, only 203 were reviewed. The remaining 167 cases were excluded for technical reasons (desiccation of smears, or broken or lost plates).

This review was done independently and in a single-blind manner regarding the final diagnosis by two cytopathologists at SITEC, a laboratory that is linked to the National Cancer Institute (Instituto Nacional de Câncer, INCA). These professionals had prior knowledge of the clinical data, when available, as is customary. The reviewed cases were reclassified in accordance with the 2001 Bethesda System and the Brazilian Nomenclature for Cytopathological Reports.^12^ The new cytological diagnoses were compared, and the two cytopathologists sought to reach a consensus regarding any disagreements. Cases for which no consensus could be reached were then reevaluated jointly with a third cytopathologist from SITEC. The cases that were thus reclassified as ASC-US and ASC-H were then included in the present study. Patients who underwent hysterectomy prior to determining the ASC cytology were excluded from the study because their cytological findings were vaginal and therefore outside of the objective of the study.

All of the patients underwent colposcopy at IFF, under supervision by an experienced colposcopist. Histological diagnoses were considered to be those obtained using material obtained by means of biopsy, large loop excision of the transformation zone (LLETZ)^22^ or conization.

The cases of CIN II/III that underwent LLETZ presented satisfactory colposcopy examinations and the squamous-columnar junction was no deeper than the first centimeter of the cervical canal. The remaining cases underwent conization.

Diagnoses obtained from histological specimens were taken to be the gold standard. When unavailable, cytocolposcopic follow-up was preferred. For the cases analyzed using histology, the more severe findings were considered to be the final diagnoses. Cases in which histological specimens were not obtained were taken to be negative outcomes (normal colposcopy or minor abnormalities). When the colposcopy examination was unsatisfactory, another cytological test was needed six months later.

Cases in which the colposcopy was unsatisfactory and there was no follow-up cytological test after six months, and cases for which we were unable to reach a diagnostic conclusion by September 2007 were considered lost from the follow-up.

## RESULTS

Through the process described above, we identified 66 cases of ASC-H and 127 of ASC-US. After allowing for exclusions and losses, our final sample was 57 cases of ASC-H and 108 of ASC-US. The age range of the individuals with a diagnosis of ASC-H was from 17 to 68 years (Figure 1).

The prevalence of CIN II/III among individuals with ASC-H cytology was 19.29% (95% confidence interval, CI: 9.05-29.55%), whereas for ASC-US the prevalence was 1.85% (95% CI: 0.0-4.64%). There were no cases of cervical neoplasia.

The risk of CIN II/III among individuals with ASC-H cytology was assessed in relation to individuals presenting ASC-US by calculating the prevalence ratio (PR). The result found was 10.42 (95% CI: 2.39-45.47%; P = 0.0002617) (Table 1).

In analyzing age groups, we used a cutoff point of 50 years of age for the women with ASC-H cytology. The prevalence of CIN II/III among the women under that age was 22.22% (10/45; 95% CI: 10.07-34.37), whereas for the older women, it was 8.33% (1/12; 95% CI: 0.0-23.97). This gave rise to a risk of 2.67 (95% CI: 0.38-18.83; P = 0.2786998) (Table 2).

**Table 1.** Risk of cervical intraepithelial neoplasia (CIN) II/III in atypical squamous cells for which high-grade squamous intraepithelial lesion cannot be ruled out (ASC-H) compared with atypical squamous cells of undetermined significance (ASC-US), calculated by means of the prevalence ratio among patients from Instituto Fernandes Figueira, Fundação Instituto Oswaldo Cruz, 1997-2007

Cytology	CIN II/III n (%)	Other diagnoses n (%)	Total
ASC-H	11 (19.30)	46 (80.70)	57
ASC-US	2 (1.85)	106 (98.15)	108
Total	13 (7.88)	152 (92.12)	165

**Table 2.** Prevalence ratio of cervical intraepithelial neoplasia (CIN) II/III according to age group of women with ASC-H cytology (Instituto Fernandes Figueira, Fundação Instituto Oswaldo Cruz, 1997-2007)

Age group	CIN II/III n (%)	Other diagnoses n (%)	Total
< 50 years	10 (22.22)	35 (77.78)	45
50 years or older	1 (8.33)	11 (91.67)	12
Total	11 (19.30)	46 (80.70)	57

## DISCUSSION

The prevalence of CIN II/III found in cases of ASC-H cytology in this study was close to 20%. However, given the sample size, its 95% CI encompassed a possible range of results from 9.05 to 29.55%. This range did not differ much from the ranges found by other authors. The differences found here may have been due to the fact that most of the authors cited here only included cases with histological diagnoses. This gives rise to higher prevalence than the reality of clinical practice, reaching values from 26% to 68%.^13^^,^^14^^,^^15^^,^^17^^,^^18^ Another likely reason was that we included cases in which the diagnostic conclusion was reached during the follow-up, which lowers the prevalence of the disease. According to Sherman et al.,^23^ the imperfect sensitivity of colposcopy for detecting small or focal pre-invasive lesions reinforces the need for strict follow-up among individuals with ASC-H cytology without lesions visible from colposcopy. Moreover, these authors stated that ALTS data suggest that samples with low cellularity may favor a cytological diagnosis of ASC-H, to the detriment of a more conclusive diagnosis of CIN II/III.

The study by McHale et al.^19^ showed a much lower prevalence of squamous intraepithelial lesions in comparison with other cited studies (12.2%; 95% CI: 8%-17%), but in consonance with our study. Also in consonance with us, these authors included cases with histological analysis and satisfactory colposcopy without lesions that were considered negative. One limitation found in their study was the high percentage of losses from follow-up (62%), with no information that might secure the selective loss. Another study in which the results were similar to ours was the one by Wang et al.,^16^ but they only considered the cases with histological results. Thus, it may be inferred that if the remaining cases had been included, the prevalence of CIN II/III would have been lower than what was found in the present study. Despite the overlapping of the 95% CI, our study may have shown greater accuracy than the other studies because part of our population was referred to colposcopy after presenting abnormalities in two cytological tests.

With the aim of enabling direct comparison with studies that only included the histological diagnoses, we recalculated the prevalence of CIN II/III in this subgroup of patients, thereby achieving the value of 28.94% (11 cases of CIN II/III among 38 patients with histological diagnoses; 95% CI: 14.52-43.36%). This prevalence comes close to what was found by authors who used this inclusion criterion and reinforces the reasons mentioned to explain our differences.

Articles published by ALTS have suggested that the risk of CIN II/III is higher among women with ASC-H cytology than with ASC-US cytology. Simsir et al.^18^ found CIN II/III prevalence of 3% among individuals with ASC-US and that the risk of pre-invasive lesions among those with ASC-H cytology was ten times greater, which is in accordance with our results. These findings show that the division of ASC cases into two groups contributed towards identifying the cases with a higher likelihood of CIN II/III, thus justifying the different procedures in terms of referral for colposcopy.

Analysis of the age groups in the studies mentioned above showed that higher prevalence of pre-invasive lesions was more frequent in younger populations. In the study by Selvaggi,^13^ the prevalence found was 68%, with an age range from 19 to 34. On the other hand, the four cases of invasive carcinoma found by Louro et al.^15^ occurred in women older than 40 years of age. Duncan and Jacob^17^ found a high prevalence of CIN II/III in postmenopausal women (46.2%), but their results were compromised by the small sample size and because the cases only had histological diagnoses. Their result differed significantly from that of Saad et al.,^21^ who found CIN II/III in 6% of the women older than 55 years of age, i.e. similar to our results for the subgroup of women 50 years of age or older. Nevertheless, when we calculated the prevalence ratio according to age group, there was no statistical significance to assert the presence of higher risk among women younger than 50 years of age, thus pointing to the possibility of a random error in the association that was found.

The fact that there were no cases of cervical cancer in this study may have been due to the small number of older women. Moreover, the occurrence of cancer in women with ASC cytology is considered rare.^24^

Further studies with larger samples and methodologies that include patients with colposcopic examinations without lesions or without histological specimens are needed in order to determine the real prevalence of CIN II/III and cancer in this group of women. The disagreement found in the results, mostly in relation to women at and beyond the menopause, indicates the need for further studies to improve the knowledge regarding how to deal with such cases.

After identifying the cases of ASCUS, there was an initial loss of 167 cases (before the review by the cytopathologists), which corresponded to 45.13%. This loss was random, due to broken and lost plates, thus hindering the new analysis and reclassification. Therefore, we believe that this loss did not interfere with the results obtained.

Adoption of the cytomorphological criteria of the 2001 Bethesda system, and the possibility of obtaining relevant information from each case probably reflects the reality of several clinical centers and laboratories in our country. Nonetheless, the discussions regarding disagreements in case diagnoses among two or three different experienced cytopathologists may have enhanced the performance in the cytopathological diagnoses of the present study.

The initial cytological tests for the present study were carried out in a single laboratory that is considered to be a reference center in Rio de Janeiro, with experienced cytopathologists and quality control. Once again, this confers reliability to the results, although it limits the possibility of generalization of the results to other populations.

Although the cytomorphological criteria used in the Brazilian nomenclature are similar to those of the 2001 Bethesda system, the terminology used in Brazil describes the subcategory ASC-US as atypical squamous cells of undetermined significance, possibly non-neoplastic.^12^ This differs from the clinical meaning of other studies, which emphasize that intraepithelial lesions cannot be ruled out.^9^ Therefore, there is a need to be careful not to make comparisons or inferences regarding the results from ASC-US cytological tests in Brazilian and international studies. In investigating Brazilian studies on cases with ASC-H cytology, we observed that even articles or recent dissertations published after the publication of the 2001 Bethesda system refer to the cytology of atypical squamous cells of undetermined significance without making the distinction between ASC-US and ASC-H. This hinders comparisons of the results found in the present study or their consideration in relation to current clinical practice.

## CONCLUSION

The prevalence of CIN II/III among patients with ASC-H cytology was significantly higher than with ASC-US. Division of ASC diagnoses into subcategories had a good capacity for distinguishing the presence of pre-invasive lesions. There were no cases of invasive carcinoma in the present study.

With regard to age groups, pre-invasive cervical lesions were more prevalent among women younger than 50 years of age. However, the risk of presenting such lesions in younger in comparison with older women did not show statistical significance.
